# Using the IL-TEM Technique to Understand the Mechanism and Improve the Durability of Platinum Cathode Catalysts for Proton-Exchange Membrane Fuel Cells

**DOI:** 10.3390/ma17061384

**Published:** 2024-03-18

**Authors:** Szymon Smykala, Barbara Liszka, Anna E. Tomiczek, Miroslawa Pawlyta

**Affiliations:** 1 Materials Research Laboratory, Faculty of Mechanical Engineering, Silesian University of Technology, Konarskiego 18A, 44-100 Gliwice, Poland; szymon.smykala@polsl.pl; 2 Faculty of Natural Sciences, University of Silesia in Katowice, Bedzinska 60, 41-200 Sosnowiec, Poland; barbara.liszka@us.edu.pl; 3 Scientific and Didactic Laboratory of Nanotechnology and Material Technologies, Faculty of Mechanical Engineering, Silesian University of Technology, Konarskiego 18A, 44-100 Gliwice, Poland; anna.tomiczek@polsl.pl

**Keywords:** proton-exchange membrane fuel cells (PEMFCs), identical-location transmission electron microscopy (IL-TEM), durability, platinum catalyst

## Abstract

Proton-exchange membrane fuel cells are one of the most promising energy conversion technologies for both automotive and stationary applications. Scientists are testing a number of solutions to increase the durability of cells, especially catalysts, which are the most expensive component. These solutions include, among others, the modification of the composition and morphology of supported nanoparticles, the platinum–support interface, and the support itself. A detailed understanding of the mechanism of platinum degradation and the subsequent improvement of the durability of the entire cell requires the development of methods for effectively monitoring the behavior of catalytic nanoparticles under various cell operating conditions. The Identical-Location Transmission Electron Microscopy (IL-TEM) method makes it possible to visually track structural and morphological changes in the catalyst directly. Because the tests are performed with a liquid electrolyte imitating a membrane, they provide better control of the degradation conditions and, consequently, facilitate the understanding of nanoparticle degradation processes in various operating conditions. This review is primarily intended to disseminate knowledge about this technique to scientists using electron microscopy in the study of energy materials and to draw attention to issues related to the characterization of the structure of carbon supports.

## 1. Introduction

Despite its enormous application potential, the commercialization process of the proton-exchange membrane fuel cell (PEMFC) is hindered by excessively high costs, low efficiency, and the insufficient durability of the most important element, platinum, which acts as a catalyst for the reactions taking place at both electrodes of the cell. To increase its efficiency and reduce its price, platinum is currently used in the form of nanoparticles evenly dispersed on the surface of a support, which is usually a nanostructured carbon material. This may partially explain the difficulties in conducting durability studies on newly developed catalysts and the discrepancies in the published results. The second source of difficulty arises from the fact that the catalyst degradation mechanism has several pathways, such as Ostwald ripening, particle migration and coalescence, particle separation, and carbon corrosion. They usually occur simultaneously, but with different contributions. The dynamics of such changes are crucial for interpreting the activity and stability of catalysts and then for designing more durable catalysts than those currently used. Therefore, a detailed understanding of the mechanism of platinum degradation and subsequent improvement in the durability of the entire cell requires the development of methods for effectively monitoring the behavior of catalytic nanoparticles in various conditions. The solution may be the IL-TEM technique, especially in combination with ex situ tests of electrochemical cells.

In traditional transmission electron microscopy (TEM) studies, the catalyst is first analyzed in a conventional manner using a rotating disc electrode (RDE). After testing, the catalyst layer (CL) is gently scraped off the RDE tip and deposited on the TEM grid. This material is compared to the fresh catalyst, enabling an understanding of the degradation mechanism as a function of the conditions used. However, this technique has the significant drawback that the tested material must be homogeneous. Otherwise, differences may lead to an inaccurate interpretation. For a correct analysis, it is necessary to compare a large number of images obtained from different parts of the sample. The IL-TEM technique involves tracing a selected fragment of the catalyst and allows scientists to use all the advantages of electron microscopy; its main benefit is that it reduces the number of images needed for correct interpretation. The TEM grid with the embedded catalyst is used as the working electrode, which allows for the repeated examination of the material and the effective monitoring of the behavior of fuel cell catalysts.

The presented review begins with a description of the importance of PEMFCs for the implementation of low-emission transport. Catalyst nanoparticles play a decisive role in the operation of the cell, but the carbon support on which they are dispersed is equally important, especially for the durability of the entire system. Due to their nanometric size, TEM is necessary to characterize the catalyst and its support with the appropriate spatial resolution. However, examining carbon materials using this technique is difficult (poor contrast, sensitivity to electron beams), and most carbon supports (e.g., Carbon Black) have been known for decades and are considered uninteresting from a scientific point of view. This leads to the omission or simplification of information regarding the structure of the carbon support (an example is the common description of Carbon Black as an amorphous material instead of a turbostratic material). Therefore, in the presented review, it was considered advisable to present nanostructured carbon materials as Pt supports in fuel cells more specifically. After explaining the importance of issues related to improving the durability of Pt on a carbon support, methods enabling the study of catalyst degradation are presented, including IL-TEM.

The results of IL-TEM studies are microscopic images showing the number, size, morphology, and arrangement of catalyst particles for subsequent stages of the experiment. It is an effective tool for comparative studies on catalyst degradation [1]. Evaluating the volume change of the available catalyst surface helps explain the degradation of fuel cell performance, further indicating its dominant mechanism [2]. An average increase in particle size (visible in the particle size distribution histogram) with a tail toward smaller particles indicates Ostwald ripening. On the other hand, the appearance of a tail toward larger particle sizes is usually the result of agglomeration. If the size distribution does not change much with degradation, detachment is considered to be the primary mechanism [3,4]. Another possible consequence of degradation is the appearance of single atoms [5,6]. The interpretation of IL-TEM images is similar to traditional TEM, but fewer images are required for analysis. Moreover, IL-TEM studies allow for more convincing information to be obtained, even if changes are not easily noticed, and thus provide an additional opportunity to develop effective methods to increase the durability of the catalysts used in PEMFCs.

In the study of Meier et al. [7], it was shown that various degradation mechanisms can occur in parallel, which makes interpretation much more difficult. Microscopic images recorded at the same location clearly showed evidence of particle migration and coalescence, particle detachment, and Pt dissolution. The authors explained the occurrence of different degradation mechanisms by local differences in the structure of the carbon support. Initially, there may have been some doubts about the occurrence of the dissolution process (no indications of platinum nanoparticle dissolution were found, for example, in the case of a high-surface-area catalyst), but soon, the dissolution of Pt was confirmed by Perez-Alonso et al. [4]. However, it should be noted that the dissolution of Pt also depends on the thickness of the CL and the scanning speed. In IL-TEM studies, the amount of catalyst is very small, which makes the probability of redeposition low. Most IL-TEM measurements use fast potential scans to shorten the measurement time, which increases the probability of Pt redeposition and consequently reduces dissolution.

Studies using the IL-TEM technique have shown that the course of degradation depends on the catalyst itself and the parameters used during measurement (atmosphere, temperature, and the rate and range of potential changes) [8]. In recent years, significant progress has been made, and currently, degradation mechanisms are effectively studied using the IL-TEM technique, both with impressive atomic resolution [3] and using 3D tomography [9]. It was also found that the measurement results are significantly influenced by differences in the structure of the carbon support, which could be modified during temperature treatment and/or Pt deposition. This clearly indicates a great potential for improving the durability of systems composed of catalytic particles deposited on a carbon support, provided that these factors are properly monitored and considered during measurements. Our review focuses on the application of IL-TEM and related methods to study the degradation of PEMFC catalysts. Unlike other publications on similar topics, more attention is paid to the description of the carbon support. It is common knowledge that the corrosion of the carbon support plays a decisive role in the high potential arising during start/stop or lack-of-fuel conditions. However, an understanding of the mechanism of platinum degradation and the subsequent improvement in durability requires supplementing the measurement procedure with information about the structure of the carbon support and its impact on the deposition of nanoparticles and their changes during the operation of the cell.

## 2. Importance of PEMFCs for Enabling Low-Carbon Transport

Humanity is faced with the need to quickly solve enormous problems related to visible and clearly dangerous climate change, a constant and rapid increase in energy consumption, and the simultaneous depletion of fossil energy sources. The consequence is either an agreement to significantly reduce the pace of economic development, or the development and dissemination of new technologies that enable energy to be obtained in a way that substantially reduces, or preferably completely eliminates, CO_2_ emissions. The second solution is more widely accepted and desired by society, and additionally, the advanced development of renewable energy technologies makes this goal seem achievable.

Some renewable energy sources have reached the appropriate technological level and are already in practical use. Among them, PEMFCs play a significant and promising role, enabling the achievement of the “carbon peak and neutrality” goals. A PEMFC is an electrochemical cell that converts the chemical energy of hydrogen into electrical energy.

The most essential advantages of PEMFCs are that it has no harmful impacts on the natural environment, high energy density, high energy conversion efficiency, and low operating temperatures [10,11]. If one compares them with other renewable energy sources, they outperform them (not only in percentage terms). The efficiency of solar power plants is only around 15–20% and is highly dependent on weather conditions. Wind power plants achieve an efficiency of 40% but require suitable weather conditions and expensive maintenance. The efficiency of hydrogen fuel cells (around 65%) significantly exceeds that of the best internal combustion engines (35–40%).

The operation of the PEMFC does not involve harmful emissions, as the only by-products of its operation are pure water and heat. The most important component of the PEMFC is the membrane electrode assembly (MEA). The cell contains an anode and a cathode, separated by a special proton-conducting and electrically insulating polymer electrolyte membrane. The gases H_2_ and O_2_ (or air) are supplied to the electrodes through microporous gas diffusion layers (GDLs). On the anode side, a hydrogen oxidation reaction (HOR) occurs, in which the hydrogen splits into protons and electrons. The protons formed migrate through the polymer membrane (usually Nafion) to the cathode. At the same time, the electrons that cannot pass through the membrane flow through an electrical circuit, generating electricity. On the cathode side, an oxygen reduction reaction (ORR) takes place: protons that have passed through the membrane and electrons from the circuit reunite with the oxygen supplied to the cathode, producing water as the only product.

PEMFCs show great potential as an energy source for vehicles and are gradually being implemented in commercial applications (Toyota, Hyundai, and Honda) [12,13,14], but PEMFCs remain less competitive than combustion engines and batteries as vehicle power sources, primarily due to their high costs and short service life. Advanced materials and developed production technologies are needed to overcome these barriers. A barrier to the further development of applications related to the use of fuel cells, particularly in fuel cell vehicles, is the high cost and limited resources of Pt. This justifies taking actions aimed at reducing the amount of Pt per unit of power of designed vehicles powered by PEMFCs and implementing a rigorous, comprehensive recycling policy: it is expected that at least 90% of the material of the fuel cells used here will be reused [15].

## 3. Nanostructured Carbon Materials as Pt Support in Fuel Cells

Three main barriers to the commercialization of PEMFCs can be identified: their price, performance, and durability. The reaction rates at the anode and cathode vary greatly. The ORR at the cathode is six or more orders of magnitude slower than the HOR at the anode, limiting efficiency. The current form of CLs in PEMFCs, of which the microstructure consists of a catalyst, a support (usually carbon), an ionomer (usually a perfluorosulfonic acid polymer), and pores, was established in the early 1990s [16]. Pt is generally used as a catalyst, and the high price of this rare noble metal has a decisive impact on the cost of the cell. Currently, most research focuses on improving catalysts and cathode electrodes. The selection and optimization of the support material also plays a significant role in this research.

**Figure 1:**
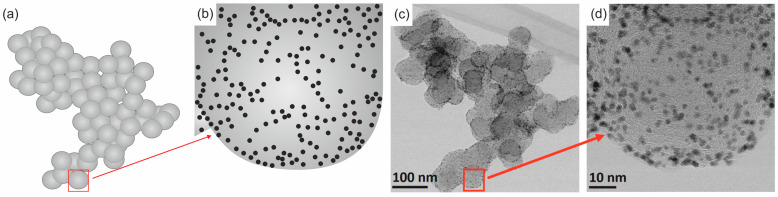
Structure of Carbon Black decorated with Pt nanoparticles: (**a**) model of a Carbon Black aggregate composed of spherical particles; (**b**) single spherical particle with Pt nanoparticles; (**c**) STEM-BF image of a Carbon Black aggregate; (**d**) an enlargement of a single spherical particle. Own materials, unpublished.

In practice, most catalysts are used as particles of a noble metal or metal compound of small (nanometric) size, evenly dispersed on the surface of a material with a large specific surface area (support) (Figure 1). The support not only maintains the metal phase in a dispersed state but also influences its catalytic activity through direct participation in each reaction step or through its interaction with the metal. The desired support properties include chemical inertness, stability under the reaction conditions, and appropriate mechanical properties, specific surface area, porosity, and physical form (the possibility of producing granulates or forms of different sizes and shapes depending on the configuration of the chemical reactor). Only a few available materials meet the complete set of desired properties and can be combined optimally. Nanostructured carbon materials are widely used to support active catalytic compounds due to their unique chemical, electrical, and mechanical surface properties, as well as the possibility of modifying their surface and structure. So far, the most crucial carbon support (from an industrial point of view) has been Carbon Black [17], the main reasons for its success being its nanostructure, availability on the market, and low price. Compared to other supports (silica, aluminum oxide, titanium oxide, or cerium oxide), the carbon surface is less reactive. Thanks to this, the structure (including the specific surface area and porosity) is less subject to changes under the influence of chemical factors. Moreover, the weak metal–carbon interaction means that metallic particles can create well-formed structures identical to those of bulk materials. Carbon Black is produced under strictly defined, repeatable industrial conditions. It is composed of three-dimensional aggregates (Figure 1a,b) consisting of multiple branched chains of complex shapes. Aggregates consist of spherical primary particles, usually less than 100 nm in diameter, which, in turn, are composed of concentrically arranged carbon layers that are parallel to each other and separated by approximately 0.335 nm (Figure 1c). Carbon Blacks can be very diverse (there are about 50 grades available on the market). The main differences concern the diameter of spherical particles, d_p_ (from several to several hundred nm), and the specific surface area, S_BET_, which usually ranges from several to over a thousand m^2^/g. Therefore, when describing the employed carbon support, it is essential, although often omitted, to provide the type and/or basic parameters (d_p_ and S_BET_) [18,19].

**Figure 2:**
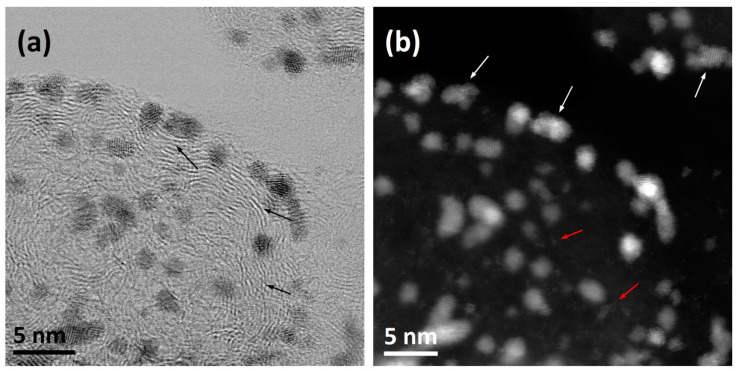
An enlargement of the single spherical particle of Carbon Black decorated with Pt nanoparticles from Figure 1b: (**a**) STEM-BF image: black arrows indicate carbon layers; (**b**) STEM-HAADF image: white arrows indicate Pt nanoparticles, and red arrows indicate atoms or clusters of Pt atoms on the carbon surface. Own materials, unpublished.

Currently, a catalyst in the form of Pt nanoparticles deposited on the Carbon Black surface can be imaged with atomic spatial resolution, thanks to the implementation of aberration correction in commercial transmission electron microscopy (TEM)/scanning TEM (STEM) [20]. Typically, two images are recorded (presented) at the same time. STEM-BF images reveal a diffraction contrast, and the arrangement of carbon layers is more visible (Figure 2a), while in HAADF images (Figure 2b), due to atomic number contrast (Z-contrast), the morphology of Pt particles is visible.

**Figure 3:**
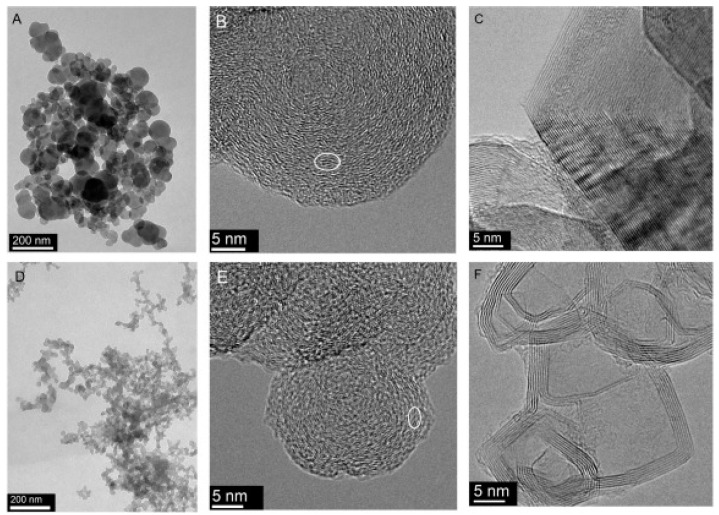
The influence of heating at high temperature (2600 °C) on the structure of two types of technical Carbon Blacks: (**A**) Printex 25—aggregate; (**B**) spherical particle not heated; (**C**) spherical particle after heating; (**D**) Color Black FW200—aggregate; (**E**) spherical particle not heated; (**F**) spherical particle after heating. TEM images [23]. The white circles in (**B**,**E**) indicate the areas, where carbon layers are arranged approximately parallel to each other.

The advantage of carbon materials, including Carbon Black, is the ability to have their structure shaped by heating at a high temperature in a protective atmosphere. During this process, the degree of crystallinity of Carbon Black increases (to a limited extent) [19]. The shape of the aggregates changes only slightly, but the shape of the particles building the aggregates changes significantly (from spherical to polyhedral), as does the organization of the carbon layers (which increase in size and are arranged approximately parallel at a constant distance from each other) [21,22]. Despite the differences, the changes in the Carbon Black structure during heating are usually similar. The degree of order in their structure is low, so they are often (wrongly) classified as amorphous materials. An example of a change in the structure of two grades of commercial Carbon Black, Printex 25 and Color Black FW200 (both manufactured by Degussa, Essen, Germany), is shown in Figure 3. The column on the left (Figure 3A,D) shows images of the entire aggregates (before heating). There are clear differences between them. Printex 25 aggregates have a compact structure and consist of large spherical particles (d_p_ = 49 nm), which are associated with a relatively low S_BET_ = 51 m^2^/g. The specific surface area of Color Black FW200 is much higher: S_BET_ = 545 m^2^/g. It forms lacy aggregates composed of small spherical particles (d_p_ = 18 nm). However, in terms of nanostructure, they are again very similar (Figure 3B,E), being composed of concentrically arranged carbon layers that are strongly defective. The size of the carbon layers arranged approximately parallel is approximately 1–2 nm, and the distance between the layers d_002_ = 0.356–0.359 nm (determined by XRD). The Carbon Black available on the market is homogeneous with regard to its chemical composition: in most cases, the level of carbon is almost 100%. These facts determine the course of the processes that take place during heating at high temperatures. The role of carbonization in this material is minor. The final degree of graphitization can be expected to be low, resulting from both the original size of the turbostratic domains and the size of the primary particles (it is not possible to form packages of carbon layers with sizes larger than the particle diameter). At the same time, HRTEM observations clearly indicate that the degree of structural order increases significantly after heating at 2600 °C (Figure 3C,F). The carbon layers straighten, elongate, and arrange themselves parallel to each other. Spherical particles change shape to polyhedral and become hollow. The size of the carbon layers arranged approximately parallel increases to approximately 6–14 nm (determined by Raman spectroscopy), while the distance between the layers d_002_ = 0.343–0.344 nm.

Thanks to the heating process, a support can be obtained that will be more corrosion-resistant, and thus, the entire catalytic system will be more durable. This process’s second advantage is better conductivity compared to non-graphitized Carbon Black. The concept of using graphitized Carbon Black to support catalytic particles appeared thirty years ago. Coloma et al. [24,25] studied the influence of the degree of support graphitization on the dispersion, stability, and catalytic activity of platinum nanoparticles deposited on soot heated in an inert gas atmosphere at temperatures up to 2200 °C. The obtained samples were characterized by specific surface area, crystal structure, and surface properties. The tests confirmed the increase in the stability of platinum nanoparticles and showed an increase in the durability of the catalyst with an increase in the degree of Carbon Black crystallinity. As expected, a higher degree of graphitization led to increased corrosion resistance, but at the same time, weakened bonds of platinum nanoparticles to the carbon surface were observed [26,27].

In recent years, there has been an increase in interest in the use of other carbon supports in PEMFCs [18,28,29,30,31,32]. Experiments are underway to use more crystalline carbon materials, primarily carbon nanotubes (CNTs) and graphene. CNTs as a platinum support should outperform conventional Carbon Black in fuel cell operating conditions in terms of durability because they provide high electrical conductivity, while specific interactions occur between Pt and CNTs. CNTs are characterized by a high chemical purity, while Carbon Black, for example, Vulcan XC-72, (Cabot Corporation, Boston, MA, USA) contains certain impurities, such as sulfur. Additionally, there are no cavities/cracks on the surfaces of CNTs, such features being dead zones for the catalysis process [33,34].

Graphene and materials based on graphene have potential advantages similar to those of CNTs as Pt supports in fuel cells [35,36,37,38]. The use of platinum nanoparticles deposited on graphene allows for an increase in both the activity and durability of fuel cells. Tests have shown that platinum on functionalized graphene has better electrochemical resistance compared to Pt particles deposited on Carbon Black. In the case of platinum nanoparticles deposited on reduced graphene oxide and graphite, aggregation is observed, because there are fewer defects in the structure of these materials compared to Carbon Black or unreduced graphene oxide. Agglomeration significantly reduces the advantageous properties of the catalyst and requires additional actions to prevent its occurrence or minimize its effects. An intensively researched solution is the creation of additional bonds involving elements other than oxygen (mainly nitrogen, sulfur, and boron) [39].

Another significant issue is the analysis of the interactions between Pt nanoparticles and carbon, which may influence catalytic performance through geometric effects and charge transfer. Doping with heteroatoms (N, B) improves the weak interactions between the carbon surface and Pt nanoparticles, increasing their activity, stability, and distribution. Carbon can also be combined with other materials (metal oxides or carbides) to form hybrid nanocomposites, which may improve the stability and increase the activity of the produced nanocomposite. Other effective and promising strategies to improve the properties of carbon supports are related to the production of materials with a core–shell structure (polyaniline (PANI)-decorated Pt/C), the deposition of catalyst nanoparticles onto N-doped porous carbon/carbon nanotubes, the use of a polymer or carbon encapsulating layer that protects and anchors Pt nanoparticles, the synthesis of Pt catalysts using ionic liquids, and the production of CD-structured electrocatalysts [32].

## 4. Importance of Durability of Pt on Carbon Support

The commercial use of PEMFCs is primarily hampered by the high cost of the Pt catalyst. The activity of the catalyst is related to the number of active centers, i.e., the share of Pt atoms located on the surface. Therefore, in order to reduce costs, the aim is to increase the surface of the Pt catalyst and use it more effectively without losing cell efficiency. This also reduces the size of the fuel cell and lowers its overall cost. The 2017 US DOE goals for catalytic converters aim to achieve a total (anode + cathode) loading of Pt or other platinum group metals of 0.125 mg cm^−2^ on MEAs capable of producing a rated stack power density of 8.0 kW g^−1^. This is a value of 8 g of metal per vehicle, similar to today’s internal combustion engine [10,40,41,42].

In addition to reducing the cathode load without loss of efficiency, research is being conducted to reduce the cathode load without losing the durability of the deposited catalyst. Long-term degradation of the CLs includes the degradation of the catalyst nanoparticles, the dissolution of the electrolyte (Nafion), and the corrosion (destruction of the structure) of the carbon support. All these phenomena cause a significant reduction in the activity of the catalyst, which is caused by changes (decrease) in the electrochemical surface area (ECSA), the loss of the electric-ion connection, or the degradation of the microstructure (porous structure) of the catalyst.

It is believed that Pt’s degradation (dissolution, detachment, and agglomeration) plays a decisive role. Platinum is known to be a thermodynamically stable element. According to the Pourbaix diagram, it dissolves at potentials higher than about 0.9 V_RHE_ at pH values lower than two, which are typical of the PEMFC cathode environment. Catalysts must survive hundreds of thousands of load cycles and tens of thousands of starts and stops during their 5000 h of stack life (during which the potential values significantly exceed safe ranges).

Pt dissolution has been experimentally confirmed by electrochemical methods combined with online detection by inductively coupled plasma mass spectrometry (ICP-MS) for a wide range of potentials, pH values, and catalyst types [43,44]. This effect is unfavorable for two reasons. Firstly, dissolved Pt can be redeposited in the catalytic layer, especially on larger nanoparticles. This effect is known as Ostwald ripening, where large particles grow at the expense of smaller ones due to differences in surface energy [45,46,47]. The result is an increase in the size of Pt nanoparticles and a simultaneous decrease in their specific surface area. Secondly, dissolved Pt can accumulate in the membrane, affecting its performance [48,49,50,51,52,53,54,55,56,57].

**Figure 4:**
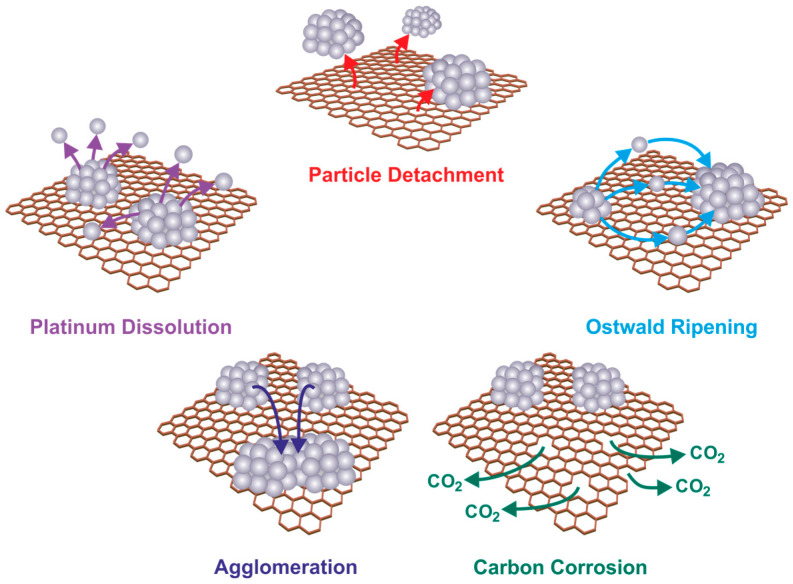
Mechanisms of degradation of platinum nanoparticles on carbon support in fuel cells [45].

The currently generally accepted mechanism of the degradation of platinum nanoparticles on a carbon support in fuel cells (Figure 4) includes the following three independent phenomena [49]:

1. The migration and agglomeration of Pt catalyst particles on the carbon support, which are the result of their nanometric size and the resulting high surface energy of the particles.
2. The dissolution of small Pt particles at high potential and their tendency to settle on larger Pt particles at low potential (Ostwald mechanism), which are caused by the difference in surface energy resulting from the inhomogeneity of the Pt particle size [50].
3. The corrosion of the carbon support as a result of a momentary high potential arising during start/stop or lack-of-fuel conditions, which causes the detachment (falling off) of Pt particles [45].

Therefore, the degradation of a Pt catalyst is a very complex process, and it is essential to recognize the difference between the so-called primary and secondary degradation of the catalyst in PEMFCs. The corrosion of the carbon support is a primary degradation phenomenon, which may result in the detachment or agglomeration of Pt particles (secondary degradation of the catalyst). Pt dissolution is a primary degradation phenomenon and is a prerequisite for subsequent degradation, for example, Ostwald ripening or Pt deposition in the membrane.

The degradation mechanisms are directly related to the interaction between the catalyst particles and the support. Strengthening the interaction between them is a potential way to improve the stability and durability of catalysts [51]. From this point of view, testing nanostructured carbon materials for better attachment/anchoring of metal nanoparticles to inhibit or mitigate the migration and agglomeration of metal particles may be an essential method to improve the durability of catalysts used in fuel cells. It should be emphasized here that this is certainly not the only possibility to increase the chances of commercialization of fuel cells from a nanoscale perspective. A wide variety of solutions are being tested around the world, including new functional semiconductor–ionic materials. The interesting scientific basis and prospects of this new technological approach are presented and discussed in [52].

## 5. Investigation Methods of Catalyst Degradation Mechanisms

Information about the state of catalytic converter degradation can be obtained during accelerated stress tests, ASTs. A symptom of Pt nanoparticles’ dissolution/reprecipitation and coalescence processes is a decrease in the ECSA [53,54]. The ECSA is widely considered to be one of the most important indicators for determining the catalytic activity of PEMFCs [55,56,57,58]. Numerous methods are used to determine the ECSA: CO gas-phase chemisorption, cyclic voltammetry (CV), and CO voltammetry—CO-stripping voltammetry. In order to investigate the degradation mechanisms of the platinum (Pt/C) catalyst on carbon, the optimal solution is to combine the AST with the monitoring of the electrochemical surface area (ECSA), oxygen reduction reaction (ORR) activities, and characterization using other techniques: X-ray photoelectron spectrometry (XPS), transmission electron microscopy (TEM), and the electrochemical impedance spectroscopy (EIS) response [59]. The EIS response is employed to monitor the resistance and capacitance change of the electrode over time and gain more insight into the carbon support corrosion mechanism. Other examples of the usefulness of the EIS technique in Pt/C research include [60,61,62].

**Figure 5:**
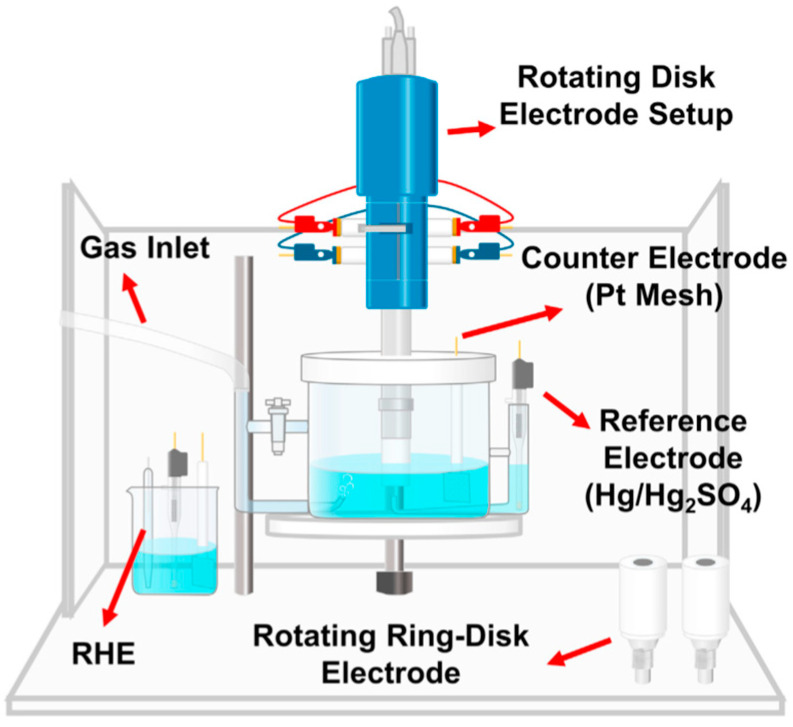
A sketch of a typical rotating electrode configuration for ORR testing [63].

**Figure 6:**
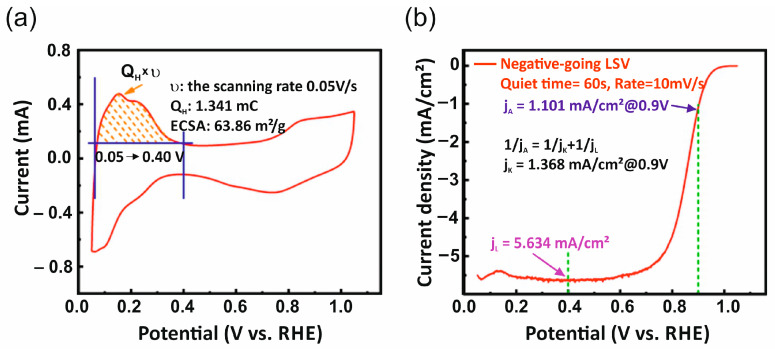
Example tests of commercial 20% Pt/C on thin-film RDE in 0.1 M HClO_4_ electrolyte: (**a**) Ar cyclic voltammetry (CV); (**b**) ORR polarization tests [63].

The RDE test is the most common method for assessing catalyst activity and its electrochemical performance (Figure 5 and Figure 6). The great advantage of this technique is measurement speed, which is why it is used as a screening method. An MEA test is more demanding, but the results are considered more reliable and correspond to the actual parameters of the tested system.

Under real-world conditions, the life of a fuel cell is expected to range from 5000 to 80,000 operating hours, depending on the specific application. Therefore, the catalyst’s condition should be assessed after such a period of time. To avoid such time-consuming measurements, special AST protocols have been developed to simulate the degradation of an entire cell or its components accurately within a reasonable time. The most commonly used AST protocol was proposed by the Fuel Cell Commercialization Conference of Japan (FCCJ) together with three car manufacturers [64]. It simulates conditions in fuel cell vehicles (FCVs) during start/stop and charging cycles. It can be used for in situ tests in MEAs and for ex situ measurements in half-cell systems. An alternative AST protocol for ex situ measurements is provided by the Department of Energy (DOE). The main differences between the FCCJ and DOE protocols are the scanning speed and dwell time used. During start/stop, the main degradation mode is attributed to the corrosion of the carbon support. While the vehicle is stationary, hydrogen gas (at the anode of the fuel cell) is gradually replaced by air. Therefore, the cathode may exhibit significantly high potentials during startup. The AST protocol, which consists of triangular potential waves ranging from 1 to 1.5 V_RHE_ with a scan rate of 500 mV s^−1^, was proposed (FCCJ) for start/stop simulation. To simulate load cycles during driving, the AST protocol was proposed using a working electrode potential in the range of 0.6 to 1 V_RHE_ [65].

AST fuel cell catalyst durability testing protocols consist of 30,000 cycles of a triangular potential sweep from 0.6 V_RHE_ to 1.0 V_RHE_ at a sweep rate of 50 mV/s. This test takes approximately 130 h, much faster than the 8000 h required for automotive applications. Additionally, an AST was developed to investigate the stability of the catalyst support. It differs from the catalyst test and consists of 5000 triangular potential sweep cycles from 1.0 V_RHE_ to 1.5 V_RHE_ at a sweep rate of 500 mV/s [66]. The target level of catalyst stability was assumed to be less than a 40% loss of initial mass activity and catalyst surface area during both ASTs. ASTs can be performed by using natural PEMFC systems (in situ) or an electrochemical cell with a liquid electrolyte imitating a membrane (ex situ). The advantage of ex situ ASTs is that they enable better control of degradation conditions and thus allow for the prediction of the life of the fuel cell while saving on material costs [66,67].

The detailed study of the surface chemistry necessary to understand the mechanism of the ORR and catalyst degradation is limited by experimental difficulties. Therefore, any opportunity to obtain any additional information is precious. Catalysts are used as nanoparticles distributed on a developed, usually porous support surface. Due to the nanometric size of the catalytic material, it becomes necessary to use TEM to study the degradation mechanisms of Pt/C catalysts under ORR conditions. A comparison of the catalysts before and after durability tests shows changes in their structure and morphology on the nanometer scale. The resulting images can show an increase in particle size, indicating that some platinum particles are bonding. Under different conditions, a systematic reduction in particle size can be observed, confirming platinum’s dissolution.

**Figure 7:**
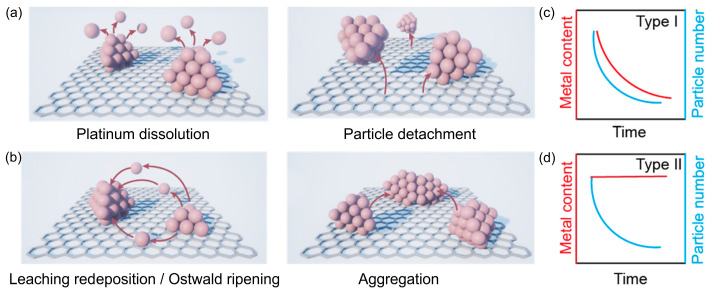
Degradation mechanisms of carbon-supported platinum nanoparticle catalysts where catalytic activity is lost as a result of: Pt dissolution and particle detachment (**a**) and Ostwald ripening and particle agglomeration (**b**). The corresponding changes in metal content and particle number (**c**,**d**) [68].

Fully understanding the behavior of catalysts in fuel cells remains a problematic research challenge, as the catalytic activity and stability are simultaneously influenced by many factors that cause several different phenomena (for example, Pt dissolution, detachment, sintering, and aggregation differ in the way that they influence the changes in the metal content and the number of nanoparticles: Figure 7). Therefore, distinguishing the mechanisms responsible for the observed changes is practically impossible. The second problem is the significant discrepancy between the actual PEMFC device and the idealized and simplified electrochemical cell explicitly designed for laboratory research.

## 6. Identical-Location Transmission Electron Microscopy (IL-TEM) Method

**Figure 8:**
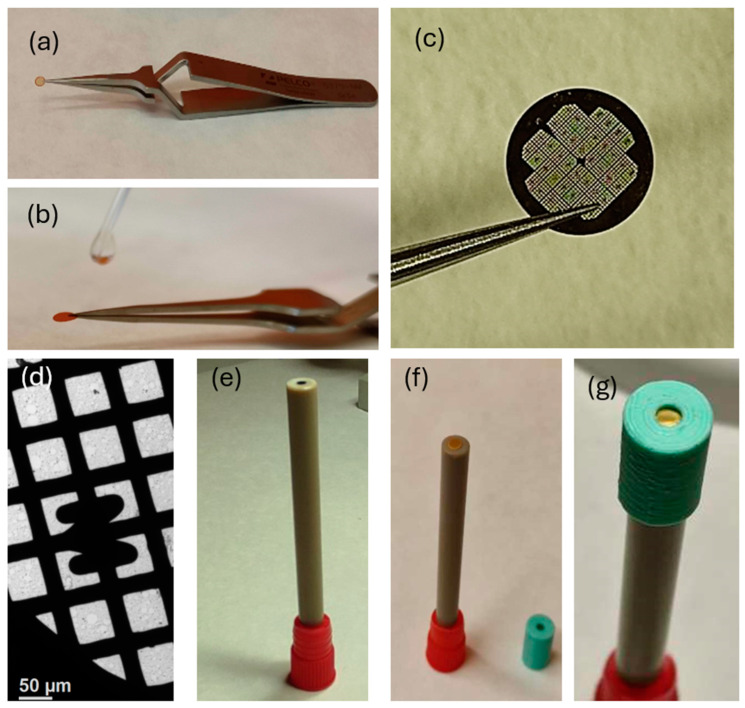
(**a**) A gold microscope grid held by tweezers; (**b**) the deposition of the alcohol-dispersed catalyst; (**c**) a view of the microscopic grid with labeled meshes; (**d**) the magnification of the microscopic mesh covered with a carbon film with embedded catalyst particles; (**e**) the working electrode; (**f**) the microscope grid applied to the electrode; (**g**) attached with a plastic cup. Own materials, unpublished.

The conventional use of TEM in studying catalyst degradation mechanisms involves comparing fresh catalyst samples and those after the reaction. TEM observations, even those with atomic spatial resolution, are performed in conjunction with ASTs. It is a powerful tool, but clear conclusions can only be drawn if the material is homogeneous, the differences are clear, and the analysis is combined with extensive statistical evaluations. These limitations do not exist in the case of the IL-TEM method, because it additionally uses imaging of selected and clearly located catalyst nanoparticles. The IL-TEM method allows scientists to examine and compare selected catalyst fragments (identical positions) before and after electrochemical aging [9,69], making it possible to visually track structural and morphological changes in the catalyst directly and facilitate the understanding of nanoparticle degradation processes under various operating conditions. The IL-TEM testing procedure involves observing the analyzed catalytic materials at the beginning of their use (BOL) and at the end of their life cycle (EOL). A small amount of the catalyst is dispersed in water or alcohol and deposited on a gold microscope grid covered with a carbon film (Figure 8a–c). In order to assess the initial state of the catalyst, microscopic images are first recorded at selected positions. The grid is then transferred to an electrochemical cell, attached with wire or tweezers, and immersed in the electrolyte as the working electrode. It can also be attached with a plastic cup (Figure 8d–g). After electrochemical processing, the TEM grid is rinsed in water, dried, and reanalyzed with the TEM microscope.

**Figure 9:**
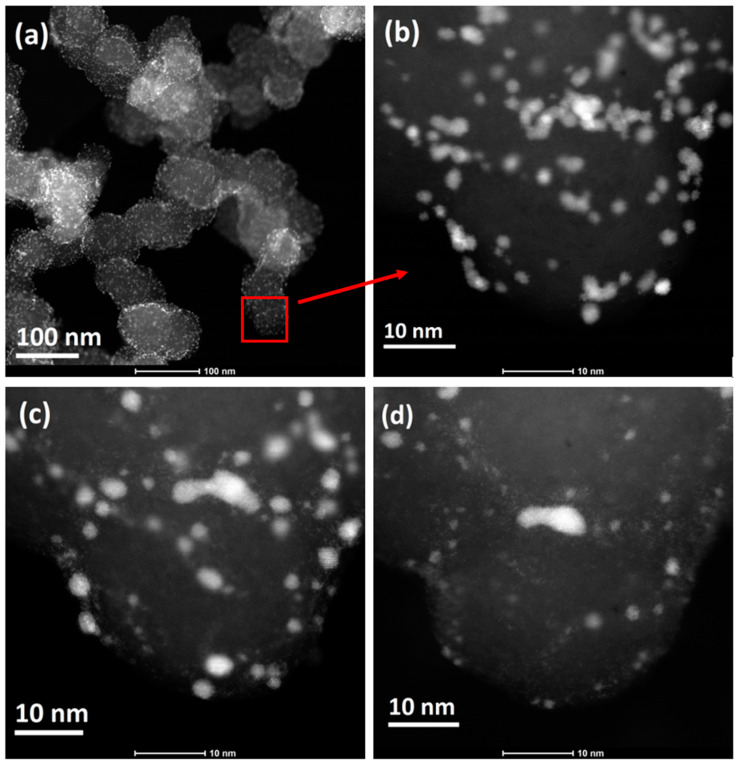
An example of the results of structural changes recorded using the IL-TEM technique. An image of a fragment of the aggregate (**a**) and a selected Carbon Black spherical particle (**b**). The same particle after 1000 (**c**) and after 5000 (**d**) potential cycles in the range of 0.6–1.1 V (HClO_4_ 0.1 M, N_2_-saturated electrolyte, at 100 mV/s). STEM-HAADF images. Own materials, unpublished.

Microscopic images allow scientists to “see” how the amount, distribution, and morphology of platinum nanoparticles change, enabling platinum changes that may be active in the reaction to be assessed and explaining which mechanism is responsible for reducing the ECSA value. Because observations can be repeated many times, it is possible to describe the dynamics of these changes. An example is shown in Figure 9, where the distribution and amount of Pt on one Carbon Black spherical particle, indicated by the red square in Figure 9a, are compared. At the beginning, Pt nanoparticles are numerous, fine, and very similar in size (Figure 9b). After 1000 cycles, the number of visible particles decreases (Figure 9c), while after 5000 cycles, only a few remain (Figure 9d). It is still difficult to clearly state whether the reduction in the amount of Pt is related to detachment or dissolution.

**Figure 10:**
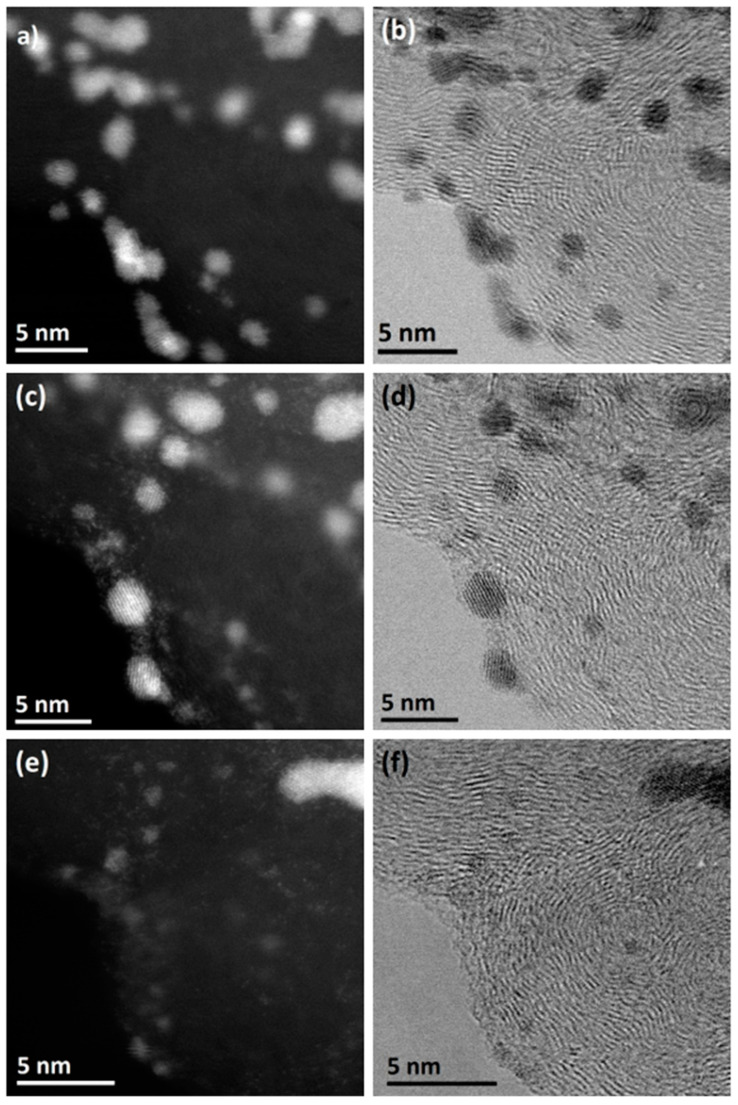
IL-TEM images taken before (**a**,**b**) and after 1000 (**c**,**d**) and 5000 (**e**,**f**) cycles of an accelerated corrosion test (the sample was subjected to 30,000 potential cycles in the range of 0.6–1.1 V (HClO_4_ 0.1 M, N_2_-saturated electrolyte, at 100 mV/s)). A fragment of the sample shown in Figure 9. STEM-HAADF (**a**,**c**,**e**) and STEM-BF (**b**,**d**,**f**) images. Own materials, unpublished.

Answering this question becomes easier after analyzing IL-TEM images obtained with better resolution (Figure 10). They represent an enlarged fragment of the images visible in Figure 10. For convenience, STEM-HAADF (left) and STEM-BF (right) images are presented simultaneously. This presentation allows for the visualization of changes (or lack thereof) in the structure of the carbon support and facilitates finding and comparing catalyst fragments at different stages of the experiment. The presented images show that after 1000 cycles, the size of some platinum particles increases (up to approx. 4 nm). At the same time, a significant number of individual Pt atoms can be seen nearby. This indicates that in the initial stage, the dominant mechanism of catalyst deactivation is Ostwald ripening. After 5000 cycles, only one large Pt particle and traces in the form of clusters of single Pt atoms are visible—evidence that the main degradation mechanism is the dissolution of platinum.

The IL-TEM technique has one unique advantage: it allows a relatively easy, cheap, and quick way to effectively test new, sometimes groundbreaking hypotheses regarding various properties, including the durability of platinum cathode catalysts for proton-exchange membrane fuel cells. The following are examples of questions that could be explored by scientists:

- Whether the modification of the structure of the carbon support affects the degradation mechanism;
- Whether Pt dissolution plays an important role in catalyst deactivation under specific conditions and correlates qualitatively with electrochemical measurements;
- Whether the minimization of surface energy during electrochemical activation affects the nanoparticle structure.

**Figure 11:**
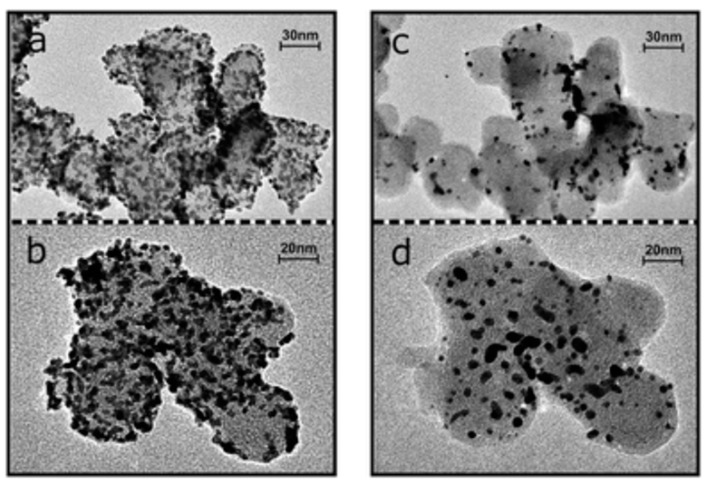
Changes in the Pt/C catalyst (unmodified support) visible in IL-TEM images taken before (**a**,**b**) and after (**c**,**d**) an accelerated corrosion test (sample was subjected to 3600 cycles between 0.4 and 1.4 V at 1 V s^−1^ in 0.1 M HClO_4_) [70].

The fact that the structure of the carbon support significantly influences the degradation mechanism was convincingly demonstrated in [70]. For this purpose, the authors compared two different carbon supports and assessed the degradation behavior using IL-TEM. Pt nanoparticles with a size of approximately 3 nm were deposited on low-surface-area (LSA) carbon. One of them was additionally modified with a transition metal. The BET surface area of LSA in both cases had a similar value of approximately 30 m^2^ g^−1^. It has been proven that a catalyst on a carbon support modified with a transition metal exhibits a much less pronounced ECSA loss than a catalyst supported on an unmodified support. After 3600 cycles, the normalized active surface area for Pt deposited on the modified carbon support decreased only to about 60%, while for the unmodified support, it was about 30%. In the second case, changes in the active surface area are perfectly visible in IL-TEM images (Figure 11). IL-TEM images proved that the modification of the carbon support could significantly reduce particle loss by ensuring better anchoring. In this way, particle detachment could be drastically reduced, and degradation was limited to a migration and coalescence or sintering mechanism, confirming the potential of stabilizing Pt particles attached to the support as a way to increase the durability of the entire catalytic system.

**Figure 12:**
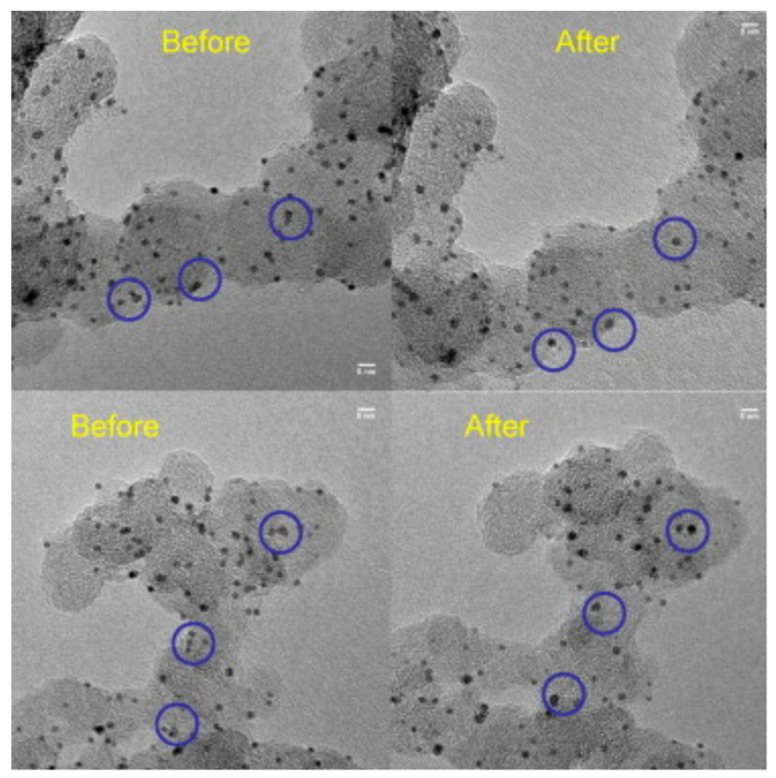
Changes in the Pt/C catalyst visible in IL-TEM images taken before and after an accelerated corrosion test (sample was subjected to 3000 cycles between 0.6 and 1.1 V at 200 mV s^−1^ in 0.1 M HClO_4_). The blue circles indicate examples of particle loss, movement, and mild sintering. Reproduced from Reference [4].

An example of IL-TEM studies showing the dissolution of Pt is the study of Perez-Alonso et al. [4], who tested a commercial catalyst with a Pt particle size of 2.3 nm (Figure 12). The sample was subjected to 3000 cycles between 0.6 and 1.1 V and between 0.6 and 1.2 V, without rotation, in an O_2_-saturated electrolyte. After ASTs, a decrease in the number and size of nanoparticles was clearly visible in IL-TEM images. A reduction in the size and the disappearance of the nanoparticles were observed, confirming that the Pt dissolved under these conditions. As a result of the ORR corrosion experiment, the volume and surface area of Pt decreased by 60% (in the case of a maximum voltage of 1.2 V) and by 8% (in the case of a maximum voltage of 1.1 V). These results correlate qualitatively with electrochemical measurements of ORR activity before and after the accelerated corrosion test, where a clear deactivation of 69 mV in the half-wave potential ΔE1/2 is observed after the accelerated corrosion test for a maximum voltage of 1.2 V and no deactivation for a maximum voltage of 1.1 V.

**Figure 13:**
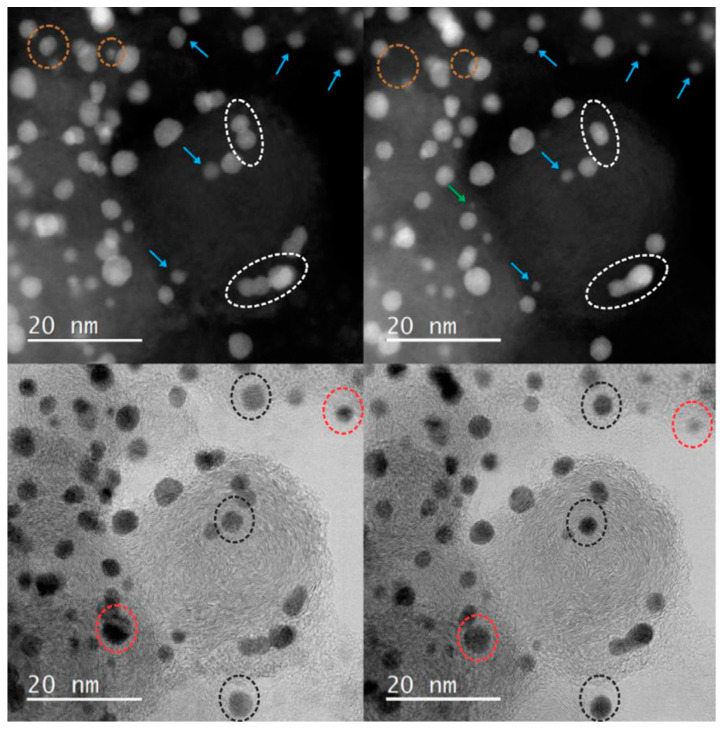
Changes in the Pt-Co/C catalyst visible in IL-TEM images taken before and after activation (the sample was subjected to 200 cycles between 0.05 and 1.2 V_RHE_ with a scan rate of 300 mV s^−1^ in 0.1 M HClO_4_). Coalescence is marked with a white dashed circles; shrinkage and reshaping with blue arrows; particle detachment with a green arrow and orange dashed circles; size reduction and Pt redistribution with red dashed circles; and rotation and Pt redistribution with black dashed circles. Reproduced from Reference [71].

**Figure 14:**
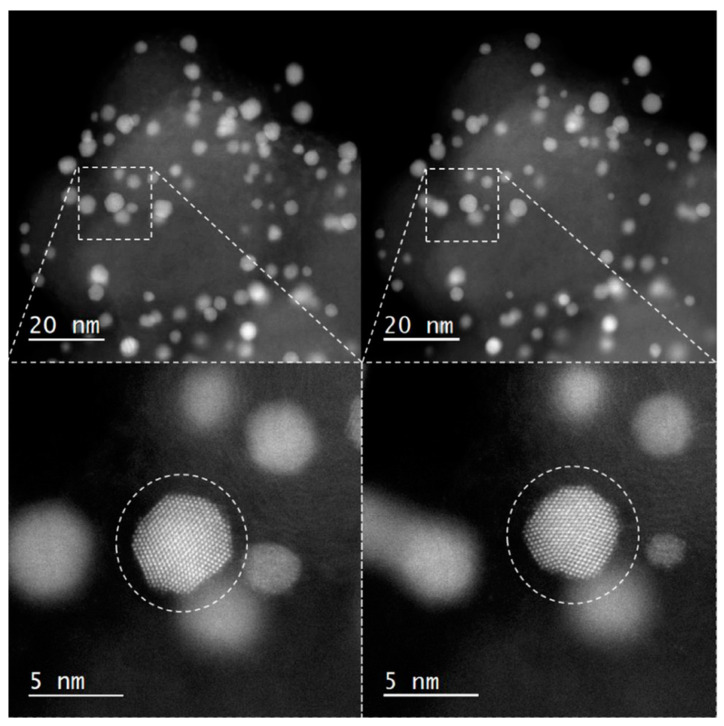
The rounding effect occurring on a Pt-Co particle after activation. IL-TEM images taken before and after activation (the sample was subjected to 200 cycles between 0.05 and 1.2 V_RHE_ with a scan rate of 300 mV s^−1^ in 0.1 M HClO_4_). Reproduced from Reference [71].

The last example shows how many different questions the IL-TEM technique can answer. Here, the atomic-scale structural changes of a benchmark industrial Pt-Co nanoalloy ORR electrocatalyst were investigated under electrochemical activation [71]. The research material was a commercially available catalyst composed of platinum cobalt alloy nanoparticles dispersed on a carbon support (Vulcan XC-72, Cabot Corporation, Boston, MA, USA). In this case, images before and after MFE measurements were compared. The sample visible in Figure 13 and Figure 14 was subjected to 200 cycles between 0.05 and 1.2 V_RHE_ with a scan rate of 300 mV s^−1^ in 0.1 M HClO_4_. Different structural changes can be observed in low-magnification images: coalescence, shrinkage and reshaping, particle detachment, size reduction, and Pt redistribution (Figure 13); however, it does not provide a full explanation of the mechanism of changes in nanoparticles. To obtain useful knowledge about the relationship between nanoparticle structure and stability, images with atomic resolution were needed. Figure 14 shows the rounding effect on the Pt-Co nanoparticle caused by the electrochemical activation protocol. This effect illustrates the minimization of surface energy during electrochemical activation.

The aim of IL-TEM research, especially in combination with ex situ methods in electrochemical cells, is to obtain information that will increase the efficiency and durability of catalysts used in PEMFCs. Images obtained for selected fragments of carbon supports with embedded Pt nanoparticles after a specific number of potential change cycles show changes in the shape and morphology of Pt nanoparticles as a result of electrochemical cycles [72,73]. The loss of total Pt volume, visible in microscopic images in the form of changes in the number and diameter of particles for precisely defined (identical) locations, can be compared with the loss of ECSA (obtained from RDE studies). In the case of measurements performed in the MEA, it is possible to correlate these discrepancies with the position of the microscopic grid inside the CL. Research can be carried out in various atmospheres (oxidizing, reducing, and neutral) [74].

Fuel cell performance declines much faster during startup/shutdown than during regular operation. This is due to the degradation of the carbon support, which leads to Pt detachment, the loss of ionomers and porosity, and the subsequent loss of performance due to mass transport limitations. Visible signs of carbon corrosion are a reduction in the distance between Pt nanoparticles due to the shrinkage of the carbon and a reduction in the surface area of the carbon. The detachment of entire fragments of aggregates is also observed [6,62]. The simulation of startup/shutdown conditions requires the use of a potential of 1.5 V. However, at a potential of 1.36 V relative to the RHE, the gold microscopic grid dissolves and redeposits on the catalyst. This is a serious difficulty during IL-TEM studies, but a solution to this problem has already been proposed by using a gold microscope grid coated with Ir [75].

Advanced IL-TEM studies (taking into account 3D observations) have shown that the mechanism of particle migration followed by coalescence operates mainly over short distances (<0.5 nm). It was observed that new particles appeared on the carbon support, which were formed as a result of the dissolution of Pt and then the formation of clusters that grew due to Ostwald ripening. The latter mechanism is also responsible for changes in the shape and growth of Pt nanoparticles, which can later result in coalescence [73].

IL-TEM has also been used to demonstrate that degradation processes using a solid are less severe compared to a liquid electrolyte, probably due to the reduced mobility of dissolved species in Nafion [76].

Serious concerns are raised by the fact that IL-TEM measurements do not sufficiently imitate the conditions of MEA measurements. In order to solve this problem, the parameters of the measurements, such as the range of potential changes, were modified, allowing the degradation process in three-electrode liquid cells to be closer to real conditions [75]. A different approach is to use the standard AST protocol and conduct measurements in a system that is as close as possible (gas environment) to the real one [73].

Table 1 shows a comparison of studies on the durability of catalysts in the form of Pt nanoparticles deposited on carbon supports for proton-exchange membrane fuel cells in which the IL-TEM technique was used. In order to compare the presented results, the main features of the catalyst used are indicated—primarily the size and loading of Pt particles and the type of carbon support. The purpose and most important result of the research are given, along with whether they were correlated with electrochemical measurements.

## 7. Summary

Due to their almost zero gas emissions, PEMFCs are one of the most promising solutions as alternative energy conversion systems to replace combustion engines. Their additional advantages include high efficiency, low maintenance costs, and high energy density. At the same time, electrode catalysts for PEMFCs are among the most demanding research topics. Understanding the mechanisms of catalyst degradation has become a major challenge in fuel cell research. Despite enormous efforts, the complex relationship between catalyst deactivation mechanisms and electrochemical degradation remains unclear due to the heterogeneous nature of the catalyst structure. Therefore, it is worth disseminating knowledge about the additional possibilities offered by the IL-TEM technique in this area.

The use of the IL-TEM technique does not require additional financial outlays. The disadvantage, however, is the time-consuming transfer of samples between the electrochemical test station and the microscope column and the high risk of sample loss in subsequent stages of the experiment. There is also controversy concerning differences in the conditions prevailing during measurements and the actual operation of the cell. Nevertheless, this technique provides a unique opportunity to characterize the phenomena occurring in PEMFCs by comparing the material structure for several sample locations before and after electrochemical cycles, during which both electrochemical analysis and electron microscopy can be used without restrictions.

Since the publication of the first IL-TEM study, significant progress has been made, and the scope of research opportunities has expanded. It has been emphasized from the beginning that such a methodology can effectively contribute to the screening of catalytic materials and the development of electrode structures to improve PEMFC technology [7]. It can be hoped that the use of IL-TEM will enable and facilitate the study of the degradation of catalysts used in PEMFCs in a more comparable and controlled manner. Currently, the main development prospects for the IL-TEM technique are related to the following:

- The establishment of a quantitative correlation with electrochemical measurements, primarily ESCA loss;
- The extension of the scope of observations made (other types of catalysts, other types of supports, other types of electrolytes, a wider range of potential changes, higher temperatures, other gas atmospheres, …);
- IL-TEM tests performed in PEMFCs under real operating conditions;
- The combination with other techniques (electrochemical and, e.g., X-ray imaging, …).

The most interesting trends in the development of identical-location microscopy include the IL-SEM method and 3D tomography. IL-SEM uses scanning electron microscopy instead of TEM [85,86]. The use of this technique improves the representativeness of the obtained results and provides insight into the catalyst degradation at different CL depths. The other promising direction of research is the use of 3D tomography, which enables the visualization of the trajectories of nanoparticles on a carbon support [8]. A special perspective on the use of IL-TEM is related to the possibility of observing phenomena occurring on the atomic scale (thanks to the use of aberration-corrected microscopes) and their direct use to interpret the operation of the catalyst.

## Figures and Tables

**Table 1:** Studies on the durability of Pt/C catalysts using IL-TEM.

Studies Focused on...	Investigated Catalyst	Results	Ref.
Size distributions, absolute number of nanoparticles, and corrosion of the carbon support	Pt/C; commercial (Tanaka Kikinzoku Kogyo K. K., Tokyo, Japan); loading of ~50 wt% Pt, particle diameter 5 nm	Degradation was observed when the sample was cycled to a high potential of 1.4 V (nanoparticle detachment). No degradation was observed when the upper potential was limited to 1.05 V or 1.2 V. There was a qualitative correlation with CO-stripping curves.	[69]
Degradation mechanisms of Pt nanoparticles deposited on carbon	Pt/C; produced in laboratory conditions; support: Carbon Black (Vulcan); 10 wt% Pt; particle diameter 2.5 nm	Catalyst degradation was observed (also for lower potential values). The degradation is mainly due to the dissolution of Pt with a minor contribution from sintering. The authors associated the increase in the dissolution fraction with the small size of the Pt catalyst nanoparticles. There was a qualitative correlation with electrochemical measurements of ORR activity.	[4]
The possibility of using IL-TEM for elevated electrolyte temperatures	Pt/C; commercial; loading of ~50 wt% Pt, particle diameter 5 nm	At elevated temperatures, the corrosion of the carbon support dominates the degradation of the catalyst. There was a qualitative correlation with ECSA loss.	[77]
The influence of the carbon support on catalyst degradation	Pt on various carbon supports (low- and high-surface-area carbon, modified by a transition metal); ~30 wt% Pt; particle diameter 3 nm	The improved properties of the catalyst with a transition-metal-modified support are due to the stabilization of the Pt particles attached to the support. Particle detachment can be drastically reduced, and the degradation is limited to a migration and coalescence or sintering mechanism. There was a qualitative correlation with ECSA loss.	[70]
Degradation mechanisms of Pt nanoparticles deposited on carbon	Pt and PtCo particles on different carbon supports (amorphous, graphitized); ~20–50 wt% Pt; particle diameter 2–5 nm	The main degradation channels are particle migration and coalescence, as well as particle detachment. No significant contribution of Pt dissolution was confirmed. Clear differences in the behavior of apparently similar catalysts during degradation were demonstrated (the great importance of the catalyst synthesis process). There was a qualitative correlation with ECSA loss.	[1]
Degradation mechanisms of Pt/C under simulated start/stop conditions	Pt/C; produced in laboratory conditions, support—Carbon Black (Vulcan); 20 wt% Pt; particle diameter 3.6 nm	Four different degradation paths were observed in one catalyst aggregate. Non-uniform degradation behavior was shown for different catalyst locations. There was a qualitative correlation with the real surface area loss.	[7]
Degradation mechanisms of Pt nanoparticles deposited on HSA carbon catalyst; investigation of the impact of different AST protocols	Pt/C; produced in laboratory conditions; support: Carbon Black (Ketjenblack EC300); 30 wt% Pt; particle diameter 2 nm	Under conditions simulating the load cycle, particle growth was mainly observed (by migration coalescence and by electrochemical Ostwald ripening). No particle detachment was observed. Under simulated start/stop conditions, particle detachment was activated as an additional degradation mechanism. There was a qualitative correlation with ECSA loss.	[78]
Degradation mechanisms of Pt/C in different potential ranges and under various gas atmospheres	Pt/C; commercial; support: Carbon Black (Vulcan); 40 wt% Pt; particle diameter 4.5 nm	The predominant degradation mechanism strongly depends on the nature of the gas atmosphere and of the upper potential limit used in accelerated stress tests. There was a qualitative correlation with ECSA loss.	[79]
Degradation mechanisms of Pt/C with various Pt loadings	Pt/C; produced in laboratory conditions; support: Carbon Blacks (Vulcan, Ketjenblack EC300); 30 wt% Pt; particle diameter 1.5–2 nm	Under conditions simulating the load cycle, no clear correlation was found between ECSA loss and the Pt:C ratio. Under conditions simulating start/stop conditions, the ECSA loss first increased with increasing Pt load and then decreased at very high loads. There was a qualitative correlation with ECSA loss.	[80]
Influence of the hydrogen evolution reaction overpotential on the mobility of Pt/C	Pt/C; commercial; particle diameter 3 nm	An increase in the number of migrating platinum particles with an increase in the overpotential value was revealed. Mechanisms have been revealed that may constitute a significant source of degradation. There was a qualitative correlation with ECSA loss.	[81]
Degradation mechanisms of Pt nanoparticles deposited on modified carbons	Pt/C modified by niobium pentoxide and tungsten carbide; produced in laboratory conditions; support: Carbon Black (Vulcan); ~20 wt% Pt; particle diameter 3–4 nm	Two phenomena leading to electrochemical surface area loss were detected: Pt particle growth and the loss of catalyst material, mainly due to the support’s degradation. There was a0 qualitative correlation with the relative surface area loss.	[82]
Experimental parameters to identify conditions that reproduce the degradation observed in MEAs	Pt and PtCo particles on various carbon supports; commercial; ~32 and 50 wt%; particle diameter 4–5 nm	By expanding the cyclic potential window, it is possible to better mimic the conditions typical of MEA measurements (the size distribution of degraded particles and the alloy composition better match those observed in MEA).	[76]
Degradation mechanisms of Pt/C, also on atomic scale	Pt/C; commercial; support: Carbon Black (Vulcan); 10 wt% Pt; particle diameter 1.5–2 nm	The degradation of the catalyst is mainly caused by the dissolution of Pt and the following secondary processes. The results suggest that the deposition of single Pt atoms on the carbon support is an important route, followed by dissolved Pt ions resulting from the dissolution of catalytic nanoparticles. There was a qualitative correlation with ECSA loss.	[5]
Degradation mechanisms of Pt/C using 3D tomography	Pt/C; commercial; support—Carbon Black (Vulcan); 5 wt% Pt; particle diameter 2 nm	The mechanism of particle migration followed by coalescence is shown to operate mainly over short distances (<0.5 nm). It is shown that new Pt particles appear on the carbon support as a result of Pt dissolution and then the formation of clusters that grow as a result of Ostwald ripening.	[9]
Quantification and visualization of Pt degradation mechanisms using correlative electron and X-ray imaging	Pt/C; commercial (Johnson Matthey Ltd., London, UK); 40 wt% Pt; particle diameter 2–5 nm.	Two types of mechanisms related to Pt degradation (with and without metal content changes) can be quantified. There was a qualitative correlation with ECSA loss.	[68]
Using iridium-coated microscopic grids (allow proper IL-TEM analysis of catalysts in durability tests without the interference of Au dissolution)	Pt/C; the alkylamine-modified Pt nanoparticle catalyst produced in laboratory conditions; support: Carbon Black (Vulcan); ~30 wt% Pt; particle diameter 2–3 nm.	It has been proven that the modification of the carbon support significantly increases the durability of the catalyst. There was a qualitative correlation with ECSA loss.	[83]
Feasibility of performing IL-TEM imaging in PEMFCs under real operating conditions (at the top of the catalytic Pt/C layer in a real PEMFC)	Pt/C; commercial; particle diameter 2–5 nm	Under conditions simulating the load cycle, Pt nanoparticles grow mainly as a result of Ostwald ripening, while the carbon support is stable. Under conditions simulating start/stop conditions, the carbon support degrades mainly through volume loss and collapse, which causes the Pt nanoparticles to approach each other, promoting additional particle growth. There was a qualitative correlation with ECSA loss.	[73]
AST protocol of potential cycles to represent the startup/shutdown settings of a fuel cell vehicle	Pt (2.3 nm) on CNT	Particle migration and coalescence are common mechanisms of Pt degradation in the early stages of the potential cycle. The mechanism of particle movement and coalescence is related to carbon corrosion, catalyzed either by Pt or by the bulk corrosion of carbon nanotubes.	[84]

## Data Availability

Datasets analyzed or generated during the study can be made available to interested scientists upon request.
